# Insulin and GLP-1 infusions demonstrate the onset of adipose-specific insulin resistance in a large fasting mammal: potential glucogenic role for GLP-1

**DOI:** 10.1002/phy2.23

**Published:** 2013-07-08

**Authors:** Jose A Viscarra, Ruben Rodriguez, Jose Pablo Vazquez-Medina, Andrew Lee, Michael S Tift, Stephen K Tavoni, Daniel E Crocker, Rudy M Ortiz

**Affiliations:** 1 School of Natural Sciences, University of CaliforniaMerced, California; 2 Scripps Institution of Oceanography, University of CaliforniaSan Diego, California; 3 Department of Biology, Sonoma State UniversityRohnert Park, California

**Keywords:** Adipose tissue, elephant seal, fatty acids, GLP-1, glucose intolerance, insulin sensitivity

## Abstract

Prolonged food deprivation increases lipid oxidation and utilization, which may contribute to the onset of the insulin resistance associated with fasting. Because insulin resistance promotes the preservation of glucose and oxidation of fat, it has been suggested to be an adaptive response to food deprivation. However, fasting mammals exhibit hypoinsulinemia, suggesting that the insulin resistance-like conditions they experience may actually result from reduced pancreatic sensitivity to glucose/capacity to secrete insulin. To determine whether fasting results in insulin resistance or in pancreatic dysfunction, we infused early- and late-fasted seals (naturally adapted to prolonged fasting) with insulin (0.065 U/kg), and a separate group of late-fasted seals with low (10 pmol/L per kg) or high (100 pmol/L per kg) dosages of glucagon-like peptide-1 (GLP-1) immediately following a glucose bolus (0.5 g/kg), and measured the systemic and cellular responses. Because GLP-1 facilitates glucose-stimulated insulin secretion, these infusions provide a method to assess pancreatic insulin-secreting capacity. Insulin infusions increased the phosphorylation of insulin receptor and Akt in adipose and muscle of early- and late-fasted seals; however, the timing of the signaling response was blunted in adipose of late-fasted seals. Despite the dose-dependent increases in insulin and increased glucose clearance (high dose), both GLP-1 dosages produced increases in plasma cortisol and glucagon, which may have contributed to the glucogenic role of GLP-1. Results suggest that fasting induces adipose-specific insulin resistance in elephant seal pups, while maintaining skeletal muscle insulin sensitivity, and therefore suggests that the onset of insulin resistance in fasting mammals is an evolved response to cope with prolonged food deprivation.

## Introduction

Insulin resistance is a common consequence of fasting (van der Crabben et al. 2008) and, although the exact mechanisms by which it manifests are still unclear, it is thought that its primary cause is the increased utilization of lipids during food deprivation (Koves et al. 2005; Samuel et al. 2010; Viscarra et al. 2012). Insulin resistance has a negative connotation due to its association with obesity and diabetes in humans, but it has been suggested to be an adaptive response to food deprivation (Viscarra et al. 2012; Tsatsoulis et al. 2013; Viscarra and Ortiz 2013). Because fasting mammals depend primarily on lipids for energy, the decreased glucose uptake and utilization resulting from impaired insulin signaling preserves the limited carbohydrate substrates for tissues that do not readily metabolize lipids (e.g., central nervous system [CNS], red blood cells [RBC]). Additionally, as less glucose is used, less protein has to be broken down for gluconeogenesis, and thus lean tissue catabolism can be reduced (Cherel et al. 1992).

Northern elephant seal pups (*Mirounga angustirostris*) undergo a 2–3 month postweaning fast during which they remain normothermic and metabolically active, while relying primarily on the oxidation of stored lipids to meet their caloric needs (Castellini et al. 1987; Adams and Costa 1993). The postweaning fasts are characterized by increased plasma free fatty acids (FFA), elevated plasma glucose, and decreased cellular insulin signaling activity (Castellini et al. 1987; Adams and Costa 1993; Champagne et al. 2005; Viscarra et al. 2011b, 2012), which collectively would constitute an insulin resistance phenotype. Furthermore, we have previously reported significant reductions in the concentrations of insulin-sensitizing hormones, adiponectin and IGF-1, along with significant increases in cortisol, which has been reported to antagonize insulin activity (Barseghian et al. 1982; Lambillotte et al. 1997; Viscarra et al. 2011a). Prolonged fasting in pups is also characterized by decreased plasma insulin and reduced glucose-stimulated insulin secretion (GSIS) (Viscarra et al. 2011a) suggesting that this period of absolute food deprivation is associated with impaired pancreatic responsiveness. However, it remains unclear whether the fasting-induced insulin resistance phenotype in fasting-adapted mammals is the result of reduced glucose tolerance by peripheral tissues, impaired/altered pancreatic responsiveness, or both.

Therefore, the present study was designed to assess the mechanisms contributing to the onset of an insulin resistant-like condition induced by prolonged food deprivation/starvation in mammals. Because elephant seals have evolved robust physiological mechanisms that have allowed them to naturally tolerate such protracted bouts of fasting, they provide an ideal model to examine the effects of fasting on insulin sensitivity. To address the hypothesis that prolonged fasting induces insulin resistance in elephant seal pups, we assessed insulin signaling in skeletal muscle and adipose tissue in response to exogenous insulin. Furthermore, we infused late-fasted animals with glucagon-like peptide 1 (GLP-1), a gastrointestinal hormone that works synergistically with glucose to enhance postprandial GSIS (MacDonald et al. 2002), to determine whether seals experience reduced pancreatic sensitivity to glucose or decreased capacity to secrete insulin late in the fast.

## Methods

All procedures were reviewed and approved by the Institutional Animal Care and Use Committees of both the University of California Merced and Sonoma State University. All work was realized under the National Marine Fisheries Service marine mammal permit #87-1743.

### Animals

Seventeen northern elephant seal pups constituting four different cohorts at Año Nuevo State Reserve were studied at two postweaning periods: early (1–2 weeks post weaning; *n* = 5) and late (6–7 weeks post weaning; *n* = 12). Pups were weighed, sedated, and infused in the field as previously described (Viscarra et al. 2011a,b). Briefly, pups were sedated with 1 mg/kg Telazol (tiletamine/zolazepam HCl, Fort Dodge Labs, Ft Dodge, IA) administered intramuscularly. Once immobilized, an 18 gauge, 3.5 inch spinal needle was inserted into the extradural vein. Blood samples were obtained, and infusions performed from this site. Continued immobilization was maintained with ∼100 mg bolus intravenous injections of ketamine as needed.

### Insulin and GLP-1 infusion protocols

Prior to each infusion protocol, preinfusion blood samples (i.v.) and tissue (adipose and muscle) biopsies were collected as previously described (Viscarra et al. 2011a). Because skeletal muscle was obtained opportunistically (attached to adipose biopsy), we were able to obtain samples from the insulin-infused animals but not the GLP-1-infused animals. All biopsies were rinsed with sterile saline, placed in cryovials, and immersed in liquid nitrogen immediately after collection as previously described (Viscarra et al. 2011a,b, 2012).

#### Insulin tolerance tests

To determine the effects of prolonged fasting on peripheral insulin activity and function, 10 fasting seal pups (early *n* = 5, late *n* = 5) were infused (i.v.) with a mass-specific dose of insulin (0.065 U/kg) (Humulin; Eli Lilly, Indianapolis, IN). Following the bolus infusion, blood samples were collected at 5, 10, 20, 30, 60, 90, and 120 min. Subsequent adipose and opportunistic muscle biopsies were collected at 60 and 120 min. Procedures were terminated at 120 min (instead of 150 min like in the GLP-GTT [glucose tolerance test] and GTT) due to concerns over the safety of the animals. Immediately following the collection of the 120 min samples, glucose was infused slowly to assist in the restoration of preinfusion levels.

#### GLP-1 + glucose tolerance tests

GLP-1 is a gastrointestinal hormone that facilitates the postprandial glucose-stimulated secretion of pancreatic insulin (MacDonald et al. 2002), and thus, its infusion should provide a method to differentiate between reduced insulin production (pancreatic capacity) and pancreatic glucose intolerance. We infused GLP-1 in the presence of glucose (GTT) to allow us to differentiate between reduced insulin production and glucose intolerance. This experimental protocol was adopted because GLP-1 in the presence of elevated glucose has the potential to provide greater insight to GLP-1-mediated effects. Seven, late-fasted seal pups were administered either a low (LDG; 10 pmol/kg; *n* = 3) or high (HDG; 100 pmol/kg; *n* = 4) dose of GLP-1 (Sigma, St Louis, MO) bolus immediately following a glucose bolus (0.5 g/kg) (i.v.) infused within 2 min. GLP1 + GTT manipulations were performed only on late-fasted animals, because we and others have demonstrated that the insulin resistance-like conditions develop with fasting duration (Houser et al. 2007; Fowler et al. 2008; Viscarra et al. 2011a,b, 2012). Following the infusions, blood samples were collected at 10, 20, 30, 60, 90, 120, and 150 min. Subsequent adipose biopsies were collected at 60 and 150 min.

#### Late-fasting GTT

To better assess and interpret the effects of GTT independent of the GLP-1 doses, we present data from late-fasted animals given the same dose of glucose (0.5 g/kg) (late GTT; same aged animals as those studied here) from our previous study (Viscarra et al. 2011a).

### Sample preparation

Blood samples were centrifuged on site for 15 min at 3000*g*, and the plasma was transferred to cryo-vials, frozen by immersion in liquid nitrogen, and stored at −80°C. Adipose and skeletal muscle were homogenized and the cytosolic- and membrane-bound protein fractions separated as previously described (Viscarra et al. 2011a). Total protein content in both cytosolic and membrane fractions was measured by the Bradford assay (Bio-Rad Laboratories, Hercules, CA) and used to normalize the loading of samples into gel wells (Viscarra et al. 2011a).

### Western blots

Twenty micrograms of total protein were resolved in 4–15% Tris-HCl SDS gradient gels. Proteins less than 100 kDa were electroblotted using the Bio-Rad Trans Blot SD semi-dry cell onto 0.45 μm nitrocellulose membranes. Proteins larger than 100 kDa were electroblotted using the Bio-Rad Mini Protean Transfer apparatus onto 0.45 μm nitrocellulose membrane. Membranes were blocked with 3% bovine serum albumin (BSA) in phosphate-buffered saline (PBS) containing 0.05% Tween 20 (PBS-T), and incubated overnight with primary antibodies. Membranes were washed, incubated with horse radish peroxidase (HRP)-conjugated secondary antibodies (SCBT, Santa Cruz, CA), rewashed, and developed by using the Pierce ECL Western Blotting Substrate (Thermo Scientific, Waltham, MA). Blots were visualized using a Kodak Image Station 440CF (Kodak Digital Sciences, Rochester, NY) and quantified using CareStream Molecular Imaging software. Because we have previously suggested that increased lipid utilization contributes to the onset of insulin resistance in late-fasted seals (Viscarra et al. 2012), we examined the following lipid handling proteins in adipose to assess the contribution of lipid metabolism to insulin signaling in late-fasted elephant seal pups: AMP kinase (AMPk; 65 kDa), adipose triglyceride lipase (ATGL; 55 kDa), fatty acid translocase (CD36; 53 kDa), fatty acid transport protein 1 (FATP1; 63 kDa), fatty acid synthase (FAS; 270 kDa), hormone-sensitive lipase (HSL; 88 kDa), lipoprotein lipase (LPL; 56 kDa), and phosphoenol pyruvate carboxy kinase-c (PEPCK-c; 67 kDa). To assess the effect of the infusions on insulin signaling we measured the phosphorylation and relative protein abundance of Akt (56 kDa), and insulin receptor (IR; 100 kDa). Primary antibodies were previously validated for use in elephant seal tissues by preadsorption with a blocking peptide to verify that the band being quantified was the band of interest and by using cell lysates recommended by the manufacturers as positive controls. In addition to consistently loading the same amount of total protein per well (25 μg), densitometry values were further normalized by correcting for the densitometry values of actin (Viscarra et al. 2011a).

### Plasma analyses

The plasma concentrations of triglycerides (TAG; Cayman Chemical, Ann Arbor, MI), glycerol (Cayman), glucose (Cayman), and nonesterified fatty acids (NEFA; Wako Chemicals; Richmond, VA) were measured with colorimetric kits. Plasma insulin (porcine insulin, Millipore, Billerica, MA), glucagon (Millipore), and cortisol (Siemens, Washington, D.C.) were measured using radioimmunoassay kits. Plasma adiponectin (canine adiponectin, Millipore) was measured using an enzyme immunoassay kit. GLP-1 (Millipore) was measured using a fluorometric assay kit. All kits (with the exception of GLP-1) have been previously validated for use with elephant seal plasma (Ortiz et al. 2001, 2003; Viscarra et al. 2011a,b, 2012). The GLP-1 assay was validated for use with elephant seal plasma by performing linearity of dilution (limit of linearity: 2–90 pmol/L) as well as spike and recovery assessments. All samples were analyzed in duplicate and run in a single assay with intraassay percent coefficient of variability of <10% for all assays.

### Adipose lipid analyses

Adipose tissue was homogenized as recommended by the manufacturer, and TAG and diacyl glycerol (DAG) were measured using commercially available kits (TAG, Cayman Chemicals; mouse DAG, CUSABIO, China). Both kits were validated by performing linearity of dilution as well as spike and recovery assessments. All samples were analyzed in duplicate and run in a single assay with intraassay, percent coefficients of variability of <10% for all assays.

### Glucose clearance calculations

For the insulin infusions, the rate of glucose disappearance (*K*) was calculated using the linear regression as the negative slope of the natural log of glucose concentrations from 0 min to 60 min postinfusion. For the GLP-1 infusions, *K* was calculated assuming that equilibration of the injected glucose with the total body pool was achieved by 20 min (Champagne et al. 2005; Fowler et al. 2008). Thus, *K* was calculated using the linear regression as the negative slope of glucose concentrations from 20 min to 150 min post infusion (Fowler et al. 2008; Viscarra et al. 2011a). This method was also used to estimate *K* from previously reported mean values (Tura et al. 2001; Wu et al. 2011; Harris and Apolzan 2012; Rodriguez et al. 2012) and compared to those calculated here in response to the infusions.

### Statistics

The baseline (or T0) measurements (plasma or tissue protein content) of the early and late insulin infusions were used to assess changes as a function of fasting duration. Postinfusion measurements were also compared between the different infusions (insulin or GLP-1). Means (±SE) were compared by analysis of variance (ANOVA) using a Fisher's PLSD post hoc test. Repeated measures ANOVA was used to determine changes following the infusions. Adipose and skeletal muscle target protein content was normalized by expressing as percent change versus the early fast mean value, and compared by ANOVA to determine changes in response to the separate infusions. Changes were considered significantly different at *P* < 0.05. Statistical analyses were performed with StatView® software (SAS Institute Inc., Cary, NC).

## Results

### Fasting reduces body mass and plasma glucose, and increases plasma lipids and adipose DAG:TAG

Mean body mass of late-fasted pups was 27% lower than early-fasted pups (Table 1). Mean plasma glucose decreased 21% with fasting (Table 1). Mean plasma TAG and NEFA increased 22% and 90%, respectively, with fasting (Table 1). Mean plasma glycerol did not change significantly with fasting; however, the NEFA to glycerol ratio increased 50% with fasting (Table 1). Fasting did not significantly change the absolute content of adipose DAG or TAG; however, adipose DAG:TAG ratio increased 55% with fasting (Table 1).

**Table 1:** Mean (±SE) body mass, plasma glucose and lipids, adipose diacylglyceride (DAG):triacylglyceride (TAG) ratio, and plasma hormones at early (2 weeks) and late (7 weeks) fasting periods from northern elephant seal pups

	Early (*n* = 5)	Late (*n* = 5)
Body mass (kg)	127 ± 1	93 ± 4^1^
Glucose (mmol/L)	9.6 ± 0.4	7.5 ± 0.4^1^
Triglycerides (mmol/L)	0.88 ± 0.09	1.07 ± 0.14^1^
Glycerol (mmol/L)	0.26 ± 0.06	0.33 ± 0.10
NEFA (mmol/L)	1.1 ± 0.1	2.1 ± 0.3^1^
NEFA:Glycerol	4.2 ± 0.3	6.3 ± 0.4^1^
Intraadipose DAG:TAG	0.45 ± 0.06	0.70 ± 0.04^1^
Adiponectin (ng/mL)	80.8 ± 3.7	65.1 ± 3.6^1^
Cortisol (nmol/L)	201 ± 13	396 ± 21^1^
Glucagon (pmol/L)	11.6 ± 1.6	12.9 ± 0.9
Glucagon-like peptide-1 (pmol/L)	4.9 ± 1.3	1.4 ± 0.9^1^
Insulin (μU/mL)	3.2 ± 0.5	2.0 ± 0.6^1^

1 Significant difference from early at *P* < 0.05.

### Fasting is associated with an insulin resistance-like endocrine profile

Mean plasma adiponectin, GLP-1, and insulin decreased 23%, 71%, and 37%, respectively, with fasting (Table 1). Mean plasma cortisol nearly doubled with fasting (Table 1). Mean plasma glucagon did not change significantly with fasting (Table 1).

### Fasting alters adipose lipid-mobilizing proteins

**Figure 1:**
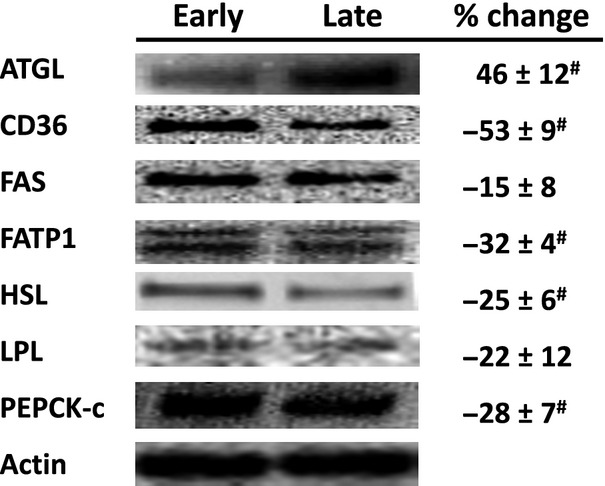
Mean (±SE) percent change of adipose lipid-mobilizing proteins from early- (2 weeks, *n* = 5) and late-fasted (7 weeks postweaning, *n* = 5) elephant seal pups. Inset: Representative Western blots for each protein. #denotes significantly (*P* < 0.05) different from early fasting. ATGL, adipose triglyceride lipase; CD36, fatty acid translocase; FAS, fatty acid synthase; FATP1, fatty acid transport protein 1; HSL, hormone-sensitive lipase; LPL, lipoprotein lipase; PEPCK-c, phosphoenol pyruvate carboxy kinase cytosolic.

Mean adipose ATGL relative protein abundance was 46% higher in late- versus early-fasted animals (Fig. 1). Conversely, relative protein abundance of adipose fatty acid transporters, CD36 and FATP1, HSL, and PEPCK-c were decreased 53%, 32%, 25%, and 28%, respectively, with fasting (Fig. 1). Neither the insulin nor the GLP-1 infusions resulted in significant time-effect changes in relative protein abundance of lipid mobilizing proteins.

### Fasting does not alter insulin-stimulated glucose clearance (*K*)

**Figure 2:**
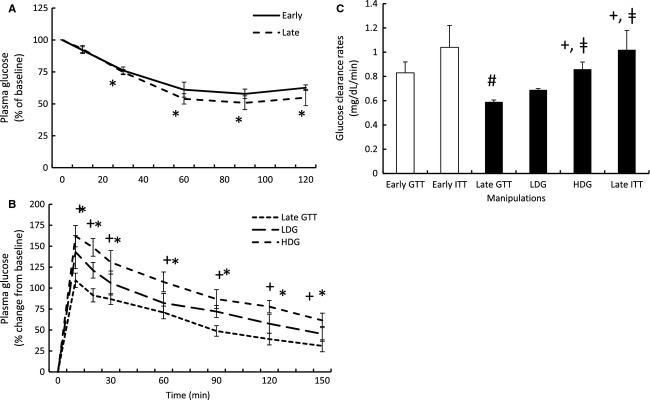
Mean (±SE) plasma glucose (A) in response to early (*n* = 5) and late insulin infusions (*n* = 5), (B) in response to low- (LDG; *n* = 3) and high-dose (HDG; *n* = 4) glucagon-like peptide-1 (GLP-1) infusions plasma (GLP-1), and (C) the resulting glucose clearance rates (*K*) in response to the exogenous infusions. #denotes significantly (*P* < 0.05) different from early fasting; *denotes significantly (*P* < 0.05) different from baseline (T0); †denotes significantly (*P* < 0.05) different from late-fasting GTT; ‡denotes significantly (*P* < 0.05) different from LDG. Late GTT glucose, and early and late glucose clearance values adapted from (Viscarra et al. 2011a).

Mean plasma glucose was reduced by 30 min and remained decreased throughout the sampling period during the insulin infusions in both the early- and late-fasted seals (Fig. 2A). A fasting effect on insulin-mediated glucose clearance was not detected (1.04 ± 0.18 vs. 1.02 ± 0.16 mg/dL per min) suggesting that peripheral tissue insulin sensitivity is not compromised with fasting duration.

### GLP-1-mediated increase in glucose clearance (*K*) is dose dependent

Peak plasma glucose was not significantly different between LDG and HDG infusions; however, HDG was associated with a 45% increase in mean plasma glucose compared with late-fasting GTT (Viscarra et al. 2011a) (161 ± 12% vs. 109 ± 8% from baseline) (Fig. 2B). In both LDG and HDG, plasma glucose remained elevated for the duration of sampling period (Fig. 2B). Glucose clearance was increased 24% with HDG compared with LDG (0.86 ± 0.06 vs. 0.69 ± 0.01 mg/dL per min) and 45% compared with late-fasting GTT (Viscarra et al. 2011a) (0.59 ± 0.02) (Fig. 2C).

### Insulin increases plasma cortisol and glucagon

**Figure 3:**
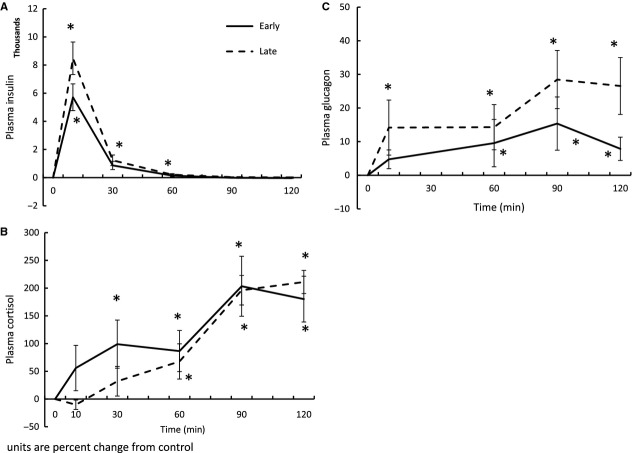
Mean (±SE) plasma (A) insulin, (B) cortisol, and (C) glucagon in response to insulin infusions in early- (2 weeks postweaning, *n* = 5) and late-(7 weeks postweaning, *n* = 5) fasted elephant seal pups. *denotes significantly (*P* < 0.05) different from baseline (T0).

Figure 3A confirms that the insulin infusions elevated circulating insulin levels for 60 min at both infusion periods (Fig. 3A). A fasting effect on insulin AUC was not detected suggesting that the metabolism of insulin is not altered with fasting (Fig. 3A). Early insulin infusion elevated mean plasma cortisol at 30 min, while the late infusion did not significantly increase cortisol until 60 min (Fig. 3B). Both early and late insulin infusions maintained cortisol elevated for the remainder of the sampling time, reaching a peak of approximately 200% (Fig. 3B) suggesting that the adrenal responsiveness to hypoglycemia is not impaired with fasting duration. Early insulin infusion elevated mean plasma glucagon at 60 min, while the late infusion elevated glucagon at 10 min, with mean circulating levels remaining elevated for the duration of the sampling period (Fig. 3C). Neither the early nor the late insulin infusions induced time-effect changes in plasma GLP-1 concentration, so only the fasting-associated changes are presented (Table 1).

### GLP-1 promotes a glucogenic endocrine response, but increases plasma insulin

**Figure 4:**
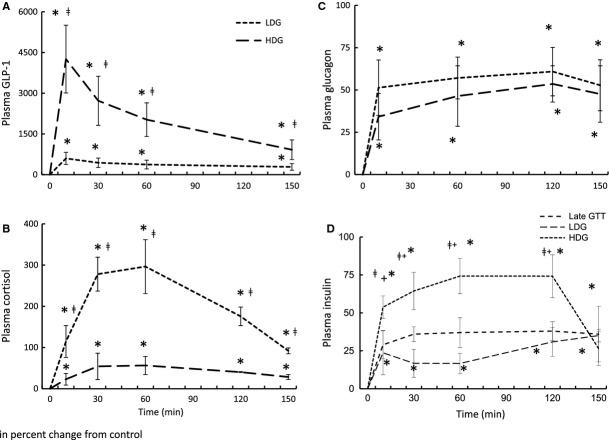
Mean (±SE) plasma (A) glucagon-like peptide-1 (GLP-1), (B) cortisol, (C) glucagon, and (D) insulin in response to low- (LDG, *n* = 3) and high-dose (HDG, *n* = 4) GLP-1 infusions in late-fasted (7 weeks postweaning) elephant seal pups. *denotes significantly (*P* < 0.05) different from baseline (T0); †denotes significantly (*P* < 0.05) different from late-fasting GTT; ‡denotes significantly (*P* < 0.05) different from LDG. Late GTT insulin values adapted from (Viscarra et al. 2011a).

Figure 4A confirms that GLP-1 infusions maintained elevated circulating GLP-1 levels throughout the sampling period and that a dose-dependent response was achieved. Both LDG and HDG induced a dose-dependent increase in mean plasma cortisol by 10 min with levels remaining elevated throughout the sampling period (Fig. 4B) despite our previous data demonstrating that GTT alone suppressed plasma cortisol within this time point suggesting that adrenal responsiveness is highly sensitive to GLP-1. HDG increased the peak plasma cortisol concentration by 430% compared with LDG (296 ± 65 vs. 56 ± 21% from baseline) (Fig. 4B). HDG and LDG increased mean plasma glucagon within 10 min with peak levels increased 51% and 34%, respectively, and remaining elevated throughout the sampling period (Fig. 4C). While a dose-dependent effect of GLP-1 on plasma glucagon was not detected, the trend is suggestive of an effect, which would imply that the pancreas is sensitive to GLP-1 levels. This is corroborated by the dose-dependent response of plasma insulin to GLP-1 (Fig. 4D). While all treatments (including late-fasting GTT) stimulated insulin secretion, the LDG infusion did not further increase the GSIS beyond the late-fasting GTT (Fig. 4D). However, HDG nearly doubled the increase in mean plasma insulin compared with LDG and late-fasting GTT (Viscarra et al. 2011a), with this increase persisting throughout the sampling period (Fig. 4D).

### Insulin and GLP-1 infusions decrease plasma lipids

#### Insulin

**Figure 5:**
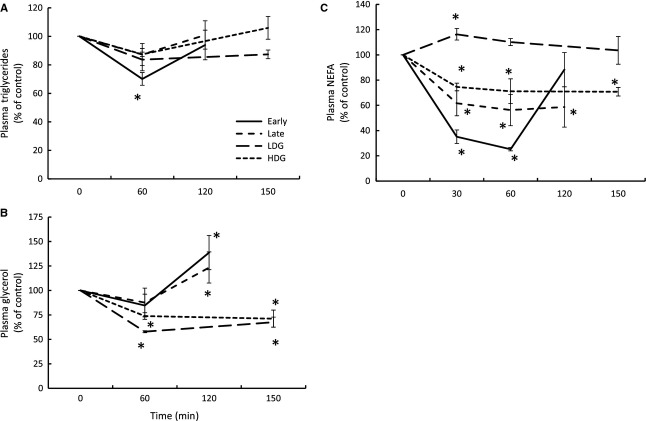
Mean (±SE) plasma (A) triglycerides, (B) glycerol, and (C) NEFA in fasting elephant seal pups in response to early (*n* = 5) and late (*n* = 5) insulin infusions and low- (LDG, *n* = 3) and high-dose (HDG, *n* = 4) GLP-1 infusions. # denotes significantly (*P* < 0.05) different from early fasting; *denotes significantly (*P* < 0.05) different from baseline (T0).

The early infusion decreased mean plasma TAG 30% at 60 min and returned to baseline levels at 120 min; however, the late infusion did not significantly change plasma TAG (Fig. 5A). Early and late infusions increased mean plasma glycerol 38% and 23%, respectively, at 120 min (Fig. 5B). The early infusion decreased mean plasma NEFA 65% at 30 min and 75% at 60 min before returning to baseline levels at 120 min, while the late infusion decreased levels 39% at 30 min, 44% at 60 min, and 42% at 120 min (Fig. 5C).

#### GLP-1 + GTT

Neither LDG nor HDG significantly changed mean plasma TAG (Fig. 5A). LDG decreased mean plasma glycerol 42% at 60 min and 33% at 150 min, and HDG decreased levels 28% at 60 min and 29% at 150 min (Fig. 5B). LDG increased mean plasma NEFA 17% at 30 min with levels returning to baseline by 60 min, while HDG decreased levels 26% at 30 min and 30% at 150 min (Fig. 5C).

### Insulin infusion stimulates insulin signaling in adipose and muscle independent of fasting duration

**Figure 6:**
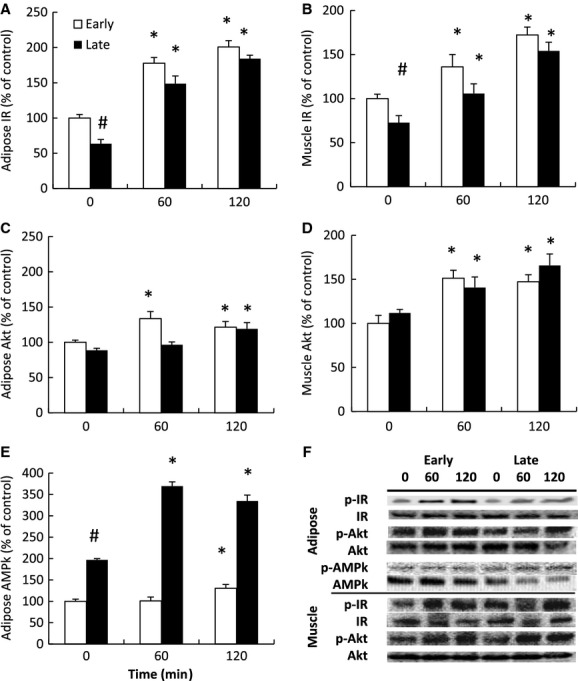
Mean (±SE) percent change of the phosphorylation of (A) adipose insulin receptor, (B) muscle insulin receptor, (C) adipose Akt, (D) muscle Akt, and (E) adipose AMP kinase (AMPk) from early- (2 weeks postweaning, *n* = 5) and late-fasted (7 weeks postweaning, *n* = 5) elephant seal pups in response to insulin infusions. (F) Representative Western blots for each protein from insulin-infused animals. #denotes significantly (*P* < 0.05) different from early fasting; *denotes significantly (*P* < 0.05) different from baseline (T0).

Fasting was associated with a 40% and 28% decrease in insulin receptor phosphorylation in adipose and muscle, respectively (Fig. 6A and B). The early insulin infusion increased mean adipose insulin receptor phosphorylation 70% at 60 min and increased to 200% at 120 min (Fig. 6A). The late insulin infusion increased adipose insulin receptor phosphorylation 134% at 60 min and 192% at 120 min (Fig. 6A). The early insulin infusion increased mean muscle insulin receptor phosphorylation 35% at 60 min and 72% at 120 min (Fig. 6B). The late insulin infusion increased muscle receptor phosphorylation 45% at 60 min and 113% at 120 min (Fig. 6B). Despite the fasting-associated difference in receptor phosphorylation (at T0), the magnitude of phosphorylation at the postinfusion periods between early and late was not different suggesting that fasting duration did not compromise insulin-mediated receptor activation.

Fasting was not associated with significant changes in either adipose or muscle Akt phosphorylation. The early insulin infusion increased mean adipose Akt phosphorylation 33% at 60 min and 21% at 120 min (Fig. 6C). The late insulin infusion increased adipose Akt phosphorylation 19% at 120 min (Fig. 6C). The early insulin infusion increased mean muscle Akt phosphorylation 51% at 60 min and 47% at 120 min, while the late infusion increased muscle Akt phosphorylation 26% at 60 min and 48% at 120 min (Fig. 6D).

Fasting was associated with a near doubling of baseline adipose AMPk phosphorylation (Fig. 6E). The early insulin infusion increased mean adipose AMPk phosphorylation 30% at 120 min, while the late infusion increased AMPk phosphorylation 88% at 60 min and 70% at 120 min (Fig. 6E).

### High-dose GLP-1 stimulates adipose insulin signaling

**Figure 7:**
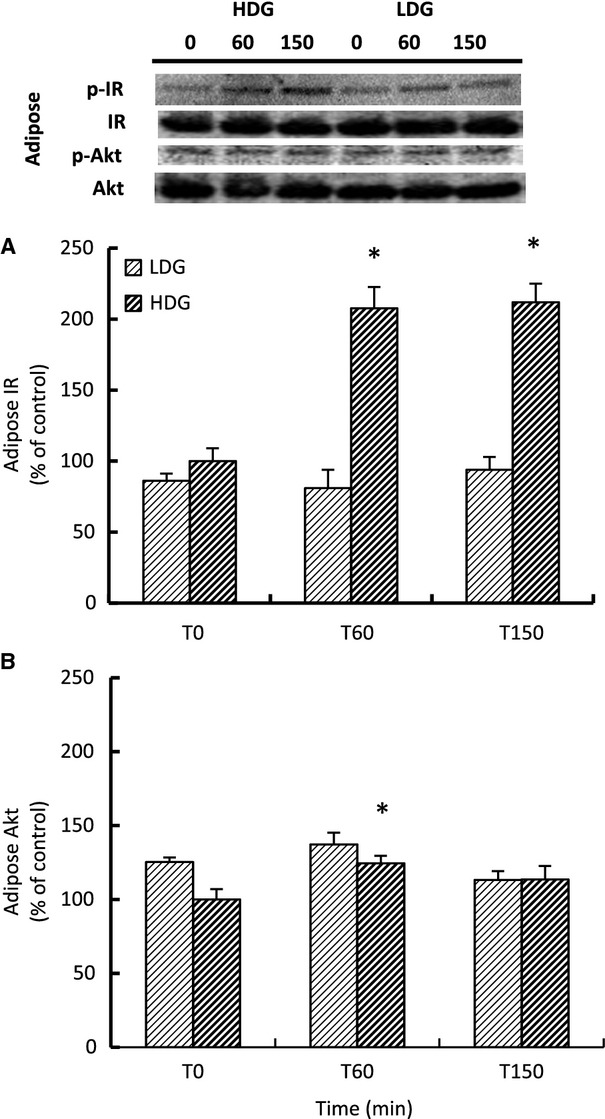
Mean (±SE) percent change of the phosphorylation of adipose (A) insulin receptor and (B) Akt in response to low (LDG; *n* = 3) and high-dose (HDG; *n* = 4) GLP-1 infusions in late-fasted (7 weeks postweaning) elephant seal pups. Inset: Representative Western blots for each protein from GLP-1 infused animals. #denotes significantly (*P* < 0.05) different from early fasting; *denotes significantly (*P* < 0.05) different from baseline (T0).

LDG did not significantly change the phosphorylation of the insulin receptor or Akt (Fig. 7A and B). HDG increased the phosphorylation of adipose insulin receptor over twofold at 60 min and remained equally elevated at 120 min, while the phosphorylation of adipose Akt increased 24% at 60 min (Fig. 7A and B).

## Discussion

This study investigated the insulin sensitivity status of fasting northern elephant seal pups by infusing different sets of animals with either GLP-1 or insulin and measuring the systemic and cellular (adipose tissue and skeletal muscle) response. Results demonstrate the onset of adipose insulin resistance in late-fasted elephant seal pups and suggest that the phenomenon manifests through the impairment of adipose insulin signaling due to the intracellular accumulation of DAG. In addition, results show that skeletal muscle remains sensitive to insulin in late-fasted elephant seal pups and the observed reductions in basal skeletal muscle insulin signaling are due primarily to decreased insulin secretion associated with decreased pancreatic glucose tolerance. Taken together, these results provide some insight as to how elephant seal pups are able to complete their development while seemingly experiencing disorders associated with metabolic deregulation and impaired lean tissue development (dyslipidemia and insulin resistance) (Pessin and Saltiel 2000; Montez et al. 2012). These results emphasize the level of metabolic control that adipose tissue possesses during the postweaning fast as it is able to elicit an insulin resistant-like state at the systemic level despite the maintenance of skeletal muscle insulin sensitivity. Additionally, results from the GLP-1 infusions suggest that GLP-1 may play a glucogenic role during fasting in addition to its role in facilitating GSIS.

Whole-body insulin resistance usually results from decreased insulin sensitivity in skeletal muscle because it is the main tissue responsible for insulin-stimulated glucose uptake (Moller et al. 1996; DeFronzo and Tripathy 2009). Therefore, maintenance of insulin sensitivity in muscle and not in adipose with fasting duration is surprising. As mentioned previously, the fasting-associated downregulation in basal insulin signaling in muscle is likely due to the hypoinsulinemia observed in late-fasted animals. This reduction in insulin secretion likely facilitates the adaptation to an insulin resistance-like condition in late-fasted seals. Nonetheless, skeletal muscle remains sensitive to insulin as the phosphorylation of its receptor and Akt increase with sampling time and is not different between early- and late-fasted animals suggesting that fasting does not compromise insulin signaling in muscle. Furthermore, each infusion (LDG, HDG, ITT) resulted in incremental increases in plasma insulin and a corresponding increase in the rate of glucose clearance suggesting that the increase in insulin signaling was functional. In addition to allowing adipose to regulate the availability of metabolic substrates in circulation, decreased insulin secretion likely serves to decrease basal glucose uptake by skeletal muscle, thereby preserving glucose for glucose-dependent tissues and maintaining the anabolic properties of insulin to facilitate the continued development of the pups during their postweaning fast.

**Figure 8:**
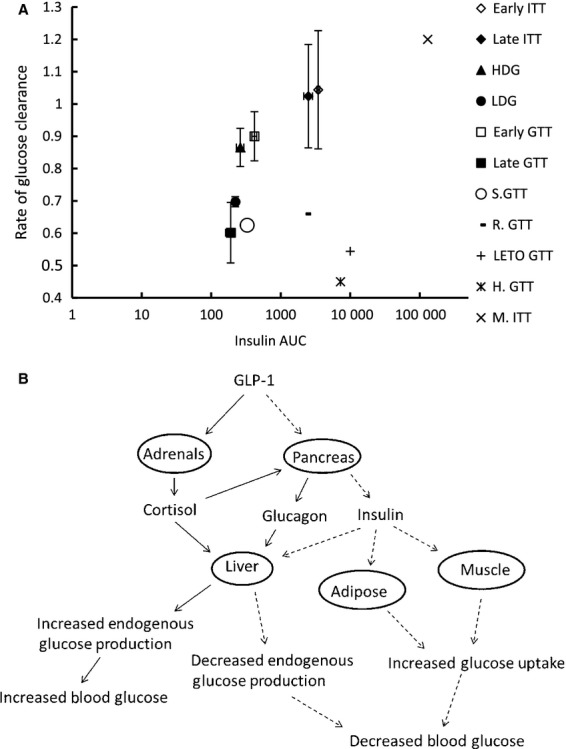
(A) Relationship between mean insulin area under the curve (AUC_insulin_) and mean glucose clearance (*K*) from the present study compared with values found in the literature for adult female elephant seals (S.GTT) (Fowler et al. 2008), Sprague-Dawley rats (R.GTT) (Harris and Apolzan 2012), Long Evans Tokushima Otsuka rats (LETO.GTT) (Rodriguez et al. 2012), humans (H.GTT) (Tura et al. 2001), and mice (M.ITT) (Wu et al. 2011). (B) Simplified schematic summarizing the effects of GLP-1 on the pancreas and adrenal glands with relation to their glucoregulatory capabilities. Solid lines are representative of steps leading to increased blood glucose, dashed lines are representative of steps leading to decreased blood glucose.

Comparing the values for glucose clearance (*K*) as a function of insulin area under the curve (AUC_insulin_) calculated from the different infusions performed here with those for other mammals (mice, rats, humans) (Fig. 8A) illustrates that seal pups do not experience the typical whole-body insulin resistance commonly associated with fasting (DeFronzo and Tripathy 2009). The relationships demonstrate that the curve for seals is shifted to the left, which suggests that a given *K* is accomplished with a smaller secretory burst of insulin (represented by AUC_insulin_) when compared with other mammals. Adult female elephant seals show a similar response to a glucose bolus (Fowler et al. 2008), suggesting that this phenotype is not exclusive to the developing pups. This would then imply that, despite an insulin resistant-like phenotype during fasting, these animals retain peripheral insulin sensitivity to a greater extent than terrestrial mammals.

Although adipose does not contribute to glucose uptake to nearly the same extent as skeletal muscle (Ferre et al. 1985), its functions as an endocrine organ (Ahima 2006) and its ability to regulate lipid availability (Viscarra and Ortiz 2013) make it a potent regulator of insulin sensitivity. Examination of adipose insulin signaling in fasting animals demonstrated a blunted insulin signaling response to an insulin infusion, consistent with the onset of insulin resistance (Pessin and Saltiel 2000) in the late-fasted seals. Similarly, the phosphorylation of insulin receptor did not result in the phosphorylation of Akt in adipose in response to the GLP-1 infusion in late-fasted animals further suggesting that prolonged food deprivation is associated with blunted insulin signaling in adipose. Furthermore, the early insulin infusion decreased plasma NEFA by more than 70%; however, the late insulin and HDG infusions only reduced their concentrations 40% and 20%, respectively, suggesting that fasting is associated with impaired insulin-mediated inhibition of lipolysis. Adipose insulin resistance is usually due to inflammation associated with obesity and the accumulation of excess lipids (Kern et al. 2001; Boden and Shulman 2002; Xu et al. 2003), but is typically not detected until it causes the loss of skeletal muscle insulin sensitivity (DeFronzo and Tripathy 2009). Therefore, the presence of adipose insulin resistance and a whole-body insulin resistance-like phenotype, while muscle insulin sensitivity is maintained in late-fasted seals is unique among mammals. These evolved mechanisms likely allowed these animals to adapt to prolonged food deprivation.

Similar to our previous findings (Viscarra et al. 2012), the NEFA:glycerol ratio in late-fasted animals increased 50% suggesting that stored TAG were not being completely metabolized. We previously suggested that the chronic activation of AMPk along with the increased relative protein abundance of adipose ATGL and reduced HSL in late-fasted animals promotes a transition to partial hydrolysis of stored triglycerides, resulting in the accumulation of intracellular DAG (Viscarra et al. 2012; Viscarra and Ortiz 2013). Additionally, the decreased relative protein abundance of fatty acid transporters, CD36 and FATP1, and the glyceroneogenic enzyme, PEPCK-c, can maintain elevated plasma NEFA while actually lipolysis decreases in late-fasted seals (Viscarra et al. 2012). Although this was suggested as a mechanism by which fasting seals can reduce the futile cycling associated with TAG metabolism, DAG accumulation may increase inflammation and potentially impair insulin signaling (Kennedy et al. 2009; Erion and Shulman 2010). Therefore, the 55% increase in the DAG:TAG ratio in late-fasted animals suggests that the DAG accumulation contributes to the blunted adipose insulin signaling.

The LDG had little effect on plasma insulin, as plasma insulin levels were similar to the levels induced by GTT alone (Viscarra et al. 2011a). However, the HDG nearly doubled the increase in plasma insulin secretion suggesting that the pancreas in these fasting-adapted mammals is sensitive to GLP-1 and the responsiveness is not impaired with fasting duration. This indicates that the fasting-induced insulin resistance and hypoinsulinemia in adapted mammals is the consequence of impaired peripheral insulin signaling and not a result of pancreatic dysfunction. More interestingly, while the HDG-mediated increase in plasma insulin was associated with an increase in the rate of glucose clearance, both the LDG and HDG increased plasma glucose compared with the GTT alone (Xu et al. 2003) suggesting that GLP-1 induced glucogenic mechanisms were sufficiently greater than insulin-mediated glucose clearance. Activation of adrenal GLP-1 receptor has been reported to increase glucocorticoid secretion (Ryan et al. 1998; Gil-Lozano et al. 2010), resulting in a subsequent increase in endogenous glucose production (Tirone and Brunicardi 2001). Therefore, the observed GLP-1 dose-dependent increase in plasma cortisol and the increase in plasma glucagon are likely responsible for the GLP-1-mediated glucogenic actions (Fig. 8B). Though it has been previously reported that cortisol has limited influence on glucose production in fasting elephant seals (Champagne et al. 2005; Houser et al. 2007), the observation of GLP-1-induced increase in plasma glucose in the presence of elevated insulin suggests that cortisol and glucagon may work synergistically to regulate glucose production during fasting. This is further demonstrated by the response to the insulin infusions, as we observe a gradual increase in both plasma cortisol and glucagon, likely in response to the rapid decrease in plasma glucose.

In conclusion, the present study demonstrated that prolonged food deprivation in the northern elephant seal pup, a large, fasting-adapted mammal, is associated with tissue-specific reductions in insulin sensitivity in which muscle insulin signaling is maintained, but adipose signaling is blunted. While late fasting is characterized by an insulin resistant-like phenotype (i.e., elevated NEFA, relatively high fasting blood glucose, etc.), the pancreas remains sensitive to GLP-1 stimulation, suggesting that the adaptation to prolonged food deprivation in these large mammals is achieved by maintaining the integrity of pancreatic function, unlike nonadapted mammals. Increased ATGL relative protein abundance and chronic AMPk activation promote partial hydrolysis of adipose TAG that results in the accumulation of DAG in late-fasted seals. Because DAG accumulation is associated with insulin resistance in rodents (Xu et al. 2003; Kennedy et al. 2009; Tsatsoulis et al. 2013), it is likely responsible for the development of blunted adipose insulin signaling in seals. The insulin resistance-like state likely assists in the regulation of metabolic substrates (Viscarra and Ortiz 2013) while permitting the continued development of postweaned pups. Despite the increase in plasma insulin and the associated increase in glucose clearance, the GLP-1-mediated increase in plasma cortisol and glucagon was sufficient to overcome these insulinogenic effects and increase plasma glucose levels. Thus, cortisol and glucagon maintain potent glucoregulatory capabilities during fasting. This glucogenic response to GLP-1 infusion suggests that GLP-1 may function as more than an insulin secretagogue and may actually be involved in regulating glucose production during fasting conditions via its effects on cortisol and glucagon. Collectively, this data provides insight to the endocrine mechanisms that regulate glucose and lipids during prolonged bouts of food deprivation in large mammals.
